# Bifid inferior turbinate with inferomedial extension: a case report and updated review of reported cases

**DOI:** 10.1093/jscr/rjag689

**Published:** 2026-08-08

**Authors:** Rehab Simsim, Abdulrahman Alhumaizi, Shahad Althubiti

**Affiliations:** Clinical Science Department, Princess Nourah bint Abdulrahman University, Airport Road, Al-Narjis District, Riyadh 84428, Saudi Arabia; Department of Otolaryngology–Head and Neck Surgery, King Abdullah bin Abdulaziz University Hospital, Airport Road, Riyadh 11564, Saudi Arabia; College of Medicine, Princess Nourah bint Abdulrahman University, Airport Road, Al-Narjis District, Riyadh 84428, Saudi Arabia

**Keywords:** bifid inferior turbinate, inferior turbinate duplication, accessory inferior turbinate, operative landmarks, case report

## Abstract

Bifid inferior turbinate is a rare anatomical variant of the lateral nasal wall that may be overlooked on nasal endoscopy or mistaken for an abnormality of the uncinate process or middle meatus. A 24-year-old woman presented with long-standing right-sided nasal obstruction, frontal headache, rhinorrhea, snoring, sleep disturbance, and allergic rhinitis. Computed tomography (CT) demonstrated an inferomedially oriented duplication of the left inferior turbinate, bilateral inferior turbinate hypertrophy, an S-shaped septal deviation, and concha bullosa. She underwent septoplasty, inferior turbinoplasty, and concha bullosa resection. Preoperative recognition of bifid inferior turbinate was important because it occurred alongside common obstructive abnormalities requiring surgery. We also provide an updated review of reported cases, focusing on the relationship with the uncinate process, anatomical orientation, and implications for surgical planning. Careful CT assessment may prevent misclassification and help preserve key endoscopic landmarks.

## Introduction

Bifid inferior turbinate (BIT), also termed duplicated or accessory inferior turbinate, is a rare variation of the lateral nasal wall in which two inferior turbinate components arise from a shared or closely related origin [1]. Bilateral BIT associated with a secondary middle turbinate has also been reported, indicating that this anomaly may coexist with other lateral nasal wall variants [2]. Selcuk *et al*. proposed that some apparent cases of BIT may represent developmental abnormalities of the uncinate process when the ipsilateral uncinate process is absent [3]. Computed tomography (CT) series have shown that uncommon inferior turbinate variants may be detected incidentally during sinonasal imaging [4]. Lee and Koh suggested that preservation of the uncinate process supports the diagnosis of true BIT rather than an uncinate process mimic [5]. We describe a young woman with inferomedial duplication of the left inferior turbinate associated with septal deviation, inferior turbinate hypertrophy and concha bullosa, and present an updated review of reported cases.

## Case report

A 24-year-old woman presented to the otolaryngology outpatient department with right-sided nasal obstruction since childhood. Associated symptoms included frontal headache, rhinorrhea, snoring, and sleep disturbance. She had a history of allergic rhinitis.

**Figure 1:**
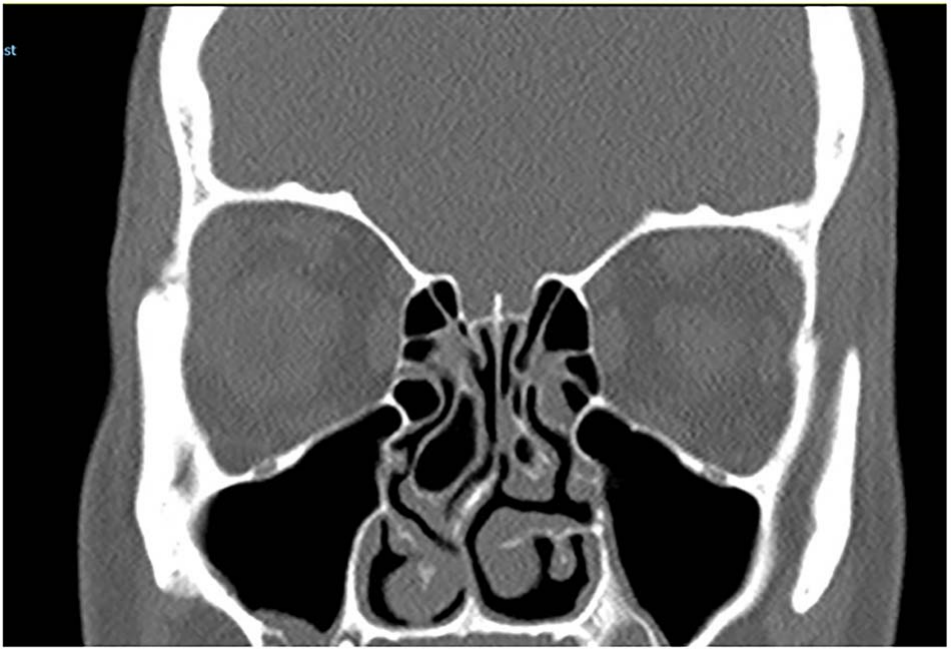
Coronal CT image of the paranasal sinuses demonstrating septal deviation, bilateral inferior turbinate hypertrophy, and inferomedial duplication of the left inferior turbinate.

Anterior rhinoscopy demonstrated an S-shaped septal deviation with minor external deformity and hypertrophy of both inferior turbinates. CT of the paranasal sinuses showed anterior septal deviation to the right and posterior deviation to the left, bilateral inferior turbinate hypertrophy, and an inferomedially arising duplication of the left inferior turbinate. Concha bullosa and mild chronic sinusitis were also present (Fig. 1).

**Figure 2:**
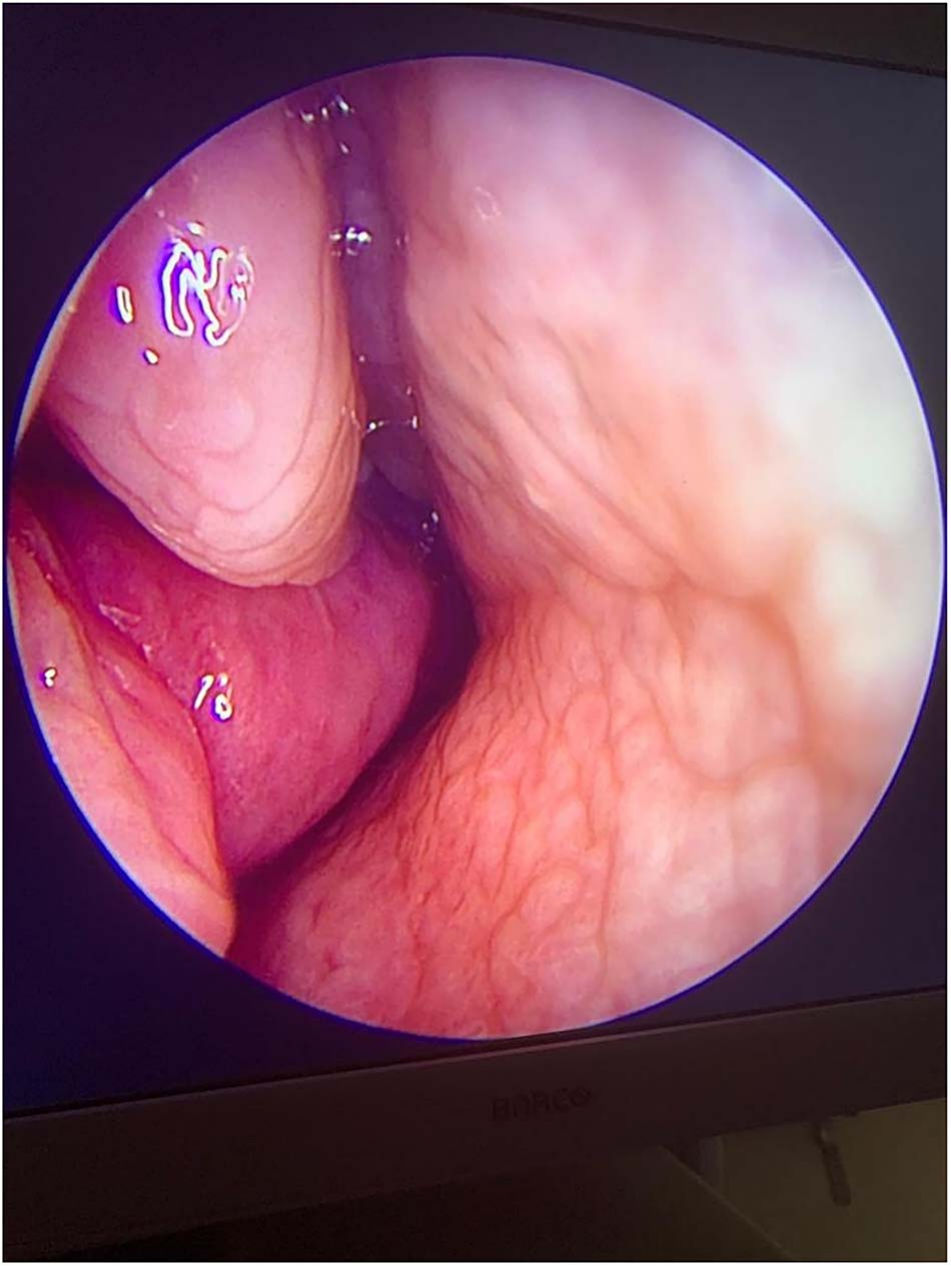
Intraoperative endoscopic view during septoplasty and inferior turbinate surgery.

The patient underwent elective septoplasty, inferior turbinoplasty, and concha bullosa resection (Fig. 2). At the latest follow-up visit, she reported mouth breathing and intermittent right-sided nasal obstruction. Nasal endoscopy showed mild residual rightward septal deviation at the nasal valve, mucosal dryness, a patent nasal airway, and appropriately reduced inferior turbinates.

## Discussion

BIT has been described using several terms, and reported cases may not represent a single anatomical process. Aksungur *et al*. reported BIT with an absent ipsilateral uncinate process, supporting a possible developmental relationship with the uncinate process [1]. In our patient, the duplicated inferior turbinate was identified preoperatively alongside common obstructive abnormalities requiring surgery. Spear *et al*. reported bilateral BIT, whereas our patient's anomaly was unilateral and extended inferomedially [2]. Selcuk *et al*. described bilateral BIT with a secondary middle turbinate and absent uncinate processes, suggesting that some cases may represent accessory or displaced lateral nasal wall structures rather than true duplication [3]. The variant should therefore be recognized without assuming that it is the primary cause of symptoms; septal deviation, turbinate hypertrophy, concha bullosa, and inflammatory disease must also be considered.

Ozcan *et al*. identified BIT in a CT series of turbinate variations, demonstrating that it may be an incidental finding [4]. Lee and Koh described true BIT with a preserved uncinate process, emphasizing the need to document the uncinate process relationship [5]. Lee *et al*. reported bilateral accessory inferior turbinates with secondary middle turbinates and absent uncinate processes, further illustrating the importance of distinguishing true BIT from uncinate process mimics [6]. Rusu *et al*. described a laterally oriented true BIT, whereas the duplicated component in our patient extended inferomedially [7]. Orientation may alter the endoscopic appearance and increase the risk of confusion with a middle-meatal or uncinate process structure. In a prospective study of 1000 patients, Vijayashre and Viswanatha identified five cases and concluded that the bifid segment generally does not require direct treatment unless it is the principal obstructive structure [8]. Our surgery therefore addressed the septum, inferior turbinate hypertrophy, and concha bullosa rather than treating the duplicated component as a primary lesion.

Recent reports reinforce the diagnostic importance of preoperative imaging. Ozbulat and Unlu showed that BIT can be identified using CT and nasal endoscopy in patients investigated for obstruction [9]. Thenata and Sensusiati reported an ipsilateral secondary middle turbinate and BIT with a preserved uncinate process, further supporting use of the uncinate process relationship to classify true BIT [10]. Duplicated middle turbinate should also be considered when lateral nasal wall anatomy is unusual because it may complicate endoscopic orientation [11]. The anatomical and radiological features of reported cases are summarized in Table 1. The value of the present case lies in the combination of an inferomedially directed variant, operative correlation, and an updated literature comparison. Careful CT assessment of the root, orientation, pneumatization, and uncinate process relationship can reduce diagnostic error, preserve surgical landmarks, and prevent unnecessary resection. Persistent postoperative symptoms should be interpreted in the context of allergic rhinitis, mucosal dryness, or nasal valve anatomy rather than attributed automatically to the variant.

**Table 1:** Reported bifid/accessory inferior turbinate cases and radiological details.

Author/year	Case context	Radiological/endoscopic details	Uncinate process/associated findings	Relevance
Aksungur *et al*., 1999 [1]	CT review of accessory nasal turbinates	Unilateral BIT with two right inferior-turbinate components sharing a root; bilateral secondary middle turbinates reported.	Ipsilateral uncinate process absent with a large maxillary ostium.	Introduced CT recognition of BIT and its possible relationship to uncinate process anomalies.
Spear *et al*., 2003 [2]	Imaging case of bilateral BIT	Bilateral bifid inferior turbinates on imaging.	Uncinate processes absent bilaterally in later literature summaries.	Supported the early view that BIT may be associated with uncinate-process absence.
Selcuk *et al*., 2008 [3]	Patient with nasal obstruction	First reported bilateral BIT with a unilateral secondary middle turbinate.	Uncinate processes missing; authors suggested BIT may represent an uncinate process developmental anomaly.	Emphasized CT assessment in symptomatic nasal obstruction.
Ozcan *et al*., 2008 [4]	CT series of nasal turbinate variations	One case of bilateral BIT among CT-evaluated patients.	Detailed uncinate process findings not the primary focus.	Demonstrated that rare inferior turbinate variants may appear incidentally in radiological series.
Lee and Koh, 2011 [5]	43-year-old man with chronic nasal obstruction	Right BIT on endoscopy and CT; septal deviation; inferior component hypertrophy.	Uncinate process present on CT.	Proposed that the term true BIT is appropriate when the uncinate process is present.
Lee *et al*., 2012 [6]	Incidental finding after facial trauma	Bilateral accessory inferior turbinates with a single soft-tissue root and bilateral secondary middle turbinates.	Uncinate process absent; authors favored accessory inferior turbinate terminology.	Important differential terminology based on embryologic origin.
Rusu *et al*., 2018 [7]	Radiological case report	True BIT described on CT/cone-beam CT; bifidity oriented laterally.	Uncinate process present.	Distinguished true BIT from false BIT/accessory inferior turbinate.
Vijayashre and Viswanatha, 2019 [8]	Prospective nasal endoscopy/CT study of 1000 patients	Five BIT cases: four unilateral, one bilateral; one pneumatized BIT.	All cases associated with absent uncinate process.	Suggested BIT usually does not need direct intervention during endoscopic sinus surgery.
Ozbulat and Unlu, 2023 [9]	Case report of rare BIT	Paranasal CT and endoscopy identified BIT as a rare cause of obstruction.	Uncinate process relationship not fully detailed in abstract-accessible text.	Recent case emphasizing CT and endoscopy before surgery.
Thenata and Sensusiati, 2024 [10]	19-year-old woman with two right nostril compartments	CT/endoscopy showed unilateral secondary middle turbinate and ipsilateral BIT; two inferior turbinates with the same root.	Ipsilateral uncinate process present.	Recent evidence supporting true BIT when the uncinate process is preserved.
Current case	24-year-old woman with long-standing obstruction	CT showed left inferomedial bifid inferior turbinate with septal deviation, bilateral inferior turbinate hypertrophy, concha bullosa, and mild chronic sinusitis.	Uncinate process relationship should be reviewed explicitly on preoperative CT.	Highlights surgical planning during septoplasty, turbinoplasty, and concha bullosa resection.

## Data Availability

All data supporting this case report are included within the article.
